# *Francisella tularensis* in Rodents, China

**DOI:** 10.3201/eid1206.051324

**Published:** 2006-06

**Authors:** Fang Zhang, Wei Liu, May C. Chu, Jun He, Qing Duan, Xiao-Ming Wu, Pan-He Zhang, Qiu-Min Zhao, Hong Yang, Zhong-Tao Xin, Wu-Chun Cao

**Affiliations:** * Beijing Institute of Microbiology and Epidemiology, Beijing, People's Republic of China;; † World Health Organization, Geneva, Switzerland;; ‡ Beijing Institute of Basic Medical Sciences, Beijing, People's Republic of China

**Keywords:** Francisella tularensis, tularemia, PCR, rodents, China, dispatch

## Abstract

A total of 420 rodents in China were examined for *Francisella tularensis* by polymerase chain reaction. The infection rates were 4.76% in total, and 11.65%, 10.00%, 6.56%, 1.77%, and 0% in Jilin, Xinjiang, Heilongjiang, Inner Mongolia, and Zhejiang, respectively. Sequence analysis showed that all the detected agents belonged to *F*. *tularensis* subsp. *holarctica*.

Tularemia is a disease caused by *Francisella tularensis*, a gram-negative, facultative intracellular bacterium. *F*. *tularensis* is generally believed to be confined to the Northern Hemisphere and has been reported in many American and Eurasian countries, for example, the United States, Mexico, Canada, Japan, the former Soviet Union, Spain, Sweden, and Norway (*1*). Terrestrial and aquatic mammals such as ground squirrels, rabbits, hares, voles, and water rats are animal reservoirs for transmission of *F*. *tularensis* to humans (*2*).

In China, *F*. *tularensis* was isolated in Daurian ground squirrels (*Spermophilus dauricus*) from Tongliao, Inner Mongolia Autonomous Region, in 1957. An outbreak of tularemia caused by contact with infected hares was first reported in Heilongjiang Province in 1959 (*3*). Thereafter, 6 cases were diagnosed in Qinghai Province in 1965 (*4*). Epidemiologic investigation identified several natural foci of the disease in Tibet from 1962 to 1972 and in Xinjiang Autonomous Region in 1986 (*5**,**6*), where *F*. *tularensis* was isolated from patients, *Ixodes liberelis* and *Dermacentor marginatus* ticks, and woolly hares (*Lepus oiostolos*). The latest outbreak occurred in 1986 at a food-processing factory in Shandong Province, where 31 of 36 workers who slaughtered hares became ill (*7*). Since then, no cases of *F*. *tularensis* infection have been reported in either humans or animal hosts. Whether the foci have become quiescent or the disease is underreported is unclear because tularemia is not a reportable disease in China. The objectives of this study were to investigate the presence of *F*. *tularensis* in rodents and to determine the subspecies type of the agent in China.

## The Study

During the spring and summer seasons of 2004 and 2005, rodents were captured with baited snap traps at 5 sites (Figure): Inner Mongolia Autonomous Region, Heilongjiang Province, Jilin Province, Xinjiang Autonomous Region, and Zhejiang Province. The first 3 sites are forested highlands in northeastern China. The study site in Xinjiang Autonomous Region is grassland in northwestern China. The study site in Zhejiang Province is wooded foothills in southern China.

**Figure:**
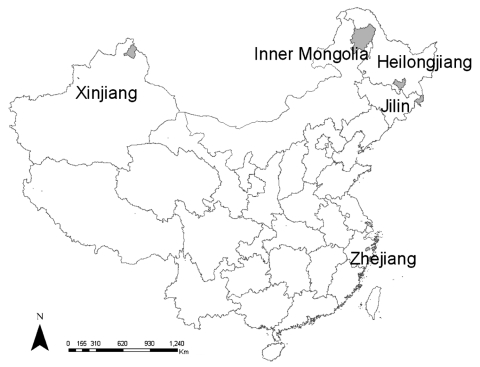
Study sites, People's Republic of China, 2004–2005.

After the species of the captured rodents was determined, a small piece of the spleen (500 mg) from each animal was used to extract DNA. Briefly, each spleen specimen was crushed with Trizol (Invitrogen, Carlsbad, CA, USA) to separate DNA from RNA after centrifugation, according to the manufacturer's instructions. Nested polymerase chain reaction (PCR) that targeted the *FopA* gene of *F*. *tularensis* was first performed as described previously (*8*). To further identify the agents in the samples by nested PCR and determine their genotype, PCR using the primer pair of C1 and C4 was performed to amplify the *ppI*-helicase region of *F*. *tularensis* gene structure (*9**,**10*). Then the products were gel purified by using Qiaquick Gel Extraction Kit (Qiagen, Hilden, Germany) and sequenced with an automated DNA sequencer (ABI Prism 377, Perkin-Elmer, Foster City, CA, USA). To minimize contamination, DNA extraction, reagent setup, amplification, and agarose gel electrophoresis were performed in separate rooms.

Of 420 rodents tested by nested PCR, 20 were positive for the *FopA* gene of *F*. *tularensis*. The overall infection rate was 4.76% with a 95% confidence interval (CI) of 2.72%–6.78%. A total of 14 species of rodents were identified in the study. Seven species, including *Apodemus peninsulae*, *A*. *agrarius*, *Cricetus migratorius*, *C. triton*, *Eutamias sibiricus*, *Meriones lybicus*, and *Clethrionomys rufocanus*, were positive for *F*. *tularensis* (Table). Although *E*. *sibiricus* and *M*. *lybicus* were most likely to be infected, with positive rates of 25% and 22.2%, respectively, no significant difference in infection rate was found among the 7 positive rodent species (χ^2^ = 11.82, degrees of freedom [*df*] = 6, p = 0.066).

**Table:** Results of detection for *Francisella tularensis* in rodents by species and geographic origin in China

Rodent species	No. positive/no. detected (%)					
	Jilin	Heilongjiang	Inner Mongolia	Xinjiang	Zhejiang	Total
*Apodemus peninsulae*	9/43 (20.93)	0/129	0/5	–*	–	9/60 (15)
*A. agrarius*	0/24	0/12	2/36 (5.56)	–	0/7 (0)	2/79 (2.5)
*Cricetus barabensis*	–	–	0/5	–	–	0/5
*Cricetus migratorius*	–	–	–	1/4 (25)	0/13(0)	1/17 (5.88)
*Cricetus triton*	1/5 (20)	–	–	–	–	1/5 (20)
*Clethrionomys rutilus*	–	–	0/13	–	–	0/13
*Clethrionomys rufocanus*	0/23	4/37(10.81)	0/2	–	–	4/62 (6.45)
*Rattus losea*	–	–	–	–	0/30	0/30
*R. norvegicus*	0/1		0/11	0/1	0/3	0/16
*R. confucianus*	–	–	–	–	0/70	0/70
*Mus musculus*	0/4(0)	–	–	0/11	–	0/15
*Microtus maximoviczii*	–	–	0/35	–	–	0/35
*Meriones lybicus*	–	–	–	1/4 (25)	–	1/4 (25)
*Eutamias sibiricus*	2/3 (66.67)	–	0/6	–	–	2/9 (22.22)
Total	12/103 (11.65)	4/61 (6.56)	2/113 (1.77)	2/20 (10)	0/123	20/420 (4.76)

*–, no rodents captured.

The prevalence of *F*. *tularensis* in rodents varied with the geographic origin (Table). The infection rate was highest in rodents in Jilin Province (11.65%), followed by Xinjiang Autonomous Region (10.00%), Heilongjiang Province (6.54%), and Inner Mongolia Autonomous Region (1.76%). No *F*. *tularensis* infection was found in rodents collected from Zhejiang Province. The geographic difference in infection rate was significant (χ^2^ = 20.91, *df* = 4, p = 0.0003). PCR assay, targeting the *ppI*-helicase region in combination with sequence analysis, identified *F*. *tularensis* in rodents in China as type B. The nucleotide sequences of the 20 positive specimens were identical to the published sequences of *F. tularensis* subsp. *holarctica* strain (GenBank accession no. AF247642.2).

## Conclusions

This study is the first PCR-based study on *F*. *tularensis* infection in rodents in China. Heilongjiang Province, Inner Mongolia, and Xinjiang Autonomous Regions were recognized as the natural foci of tularemia ≈40 years ago. The high prevalence of *F*. *tularensis* infection in rodents indicates that tularemia natural foci still exist in these areas. *F*. *tularensis* was detected for the first time in Jilin Province, which borders Heilongjiang Province and had similar landscape characteristics. Whether this newly described natural focus in Jilin Province is associated with human infection should be further investigated. In any case, this finding demonstrates that *F. tularensis* seems to be distributed more widely in China than expected. The extensive presence of *F*. *tularensis* indicates a potential threat to human health. The fact that rodents trapped from Zhejiang Province (southern China) were negative for the bacterium verifies our belief that *F*. *tularensis* is present only in northern China. The geographic variation in infection rate is likely attributable to the difference in biologic characteristics of each study site or a selection bias. Further studies are needed to clarify this question.

Rodents are efficient natural reservoirs for *F*. *tularensis* (*2**,**11*). In recent years, reports on human tularemia transmitted from small animals have been increasing (*12*). In this study, several rodent species were found to harbor *F*. *tularensis*, but which species are the main hosts in China is still unknown because no significant difference in infection rate was observed among rodents, regardless of their geographic origin. Systematic epidemiologic studies are required to investigate characteristics of natural foci and the role of both wild and domestic animals in transmission of *F*. *tularensis* to humans.
